# Sulphur in the Treatment of Pityriasis Capitis

**Published:** 1894-03

**Authors:** Bernard Wolff

**Affiliations:** Lecturer on Diseases of the Skin in the Southern Medical College; Atlanta, Ga.; 56½ Whitheall Street


### Southern Medical Record.

A MONTHLY JOURNAL OF MEDICINE AND SURGERY.

VOL. XXIV. ATLANTA, GA., MARCH, 1894. No. 3.

### Original Articles.

### SULPHUR IN THE TREATMENT OF PITYRIASIS CAPITIS.

BY BERNARD WOLFF, M. D.,

Lecturer on Diseases of the Skin in the Southern Medical College.

ATLANTA, GA.

It is not often that general practitioners concern themselves with the treatment of this cutaneous affection, from the reason, perhaps, that the evils resulting from it are apparently slight and from its sluggish response to therapeutic procedures.

It has, however, since the assertion by Unna, in 1887, of the identity of pityriasis capitis (alopecia pityroides of Pincus, seborrhea sicca) with seborrheic eczema, gained a new importance and a fresh interest, and as a cause, and indeed the most frequent cause, of premature baldness regularly engages the attention of the dermatologist. Unna considers the scalp as the brooding ground of the micro-organisms (morococci and flask bacilli or Melassez's spores) of seborrheic eczema; that here they find the best conditions for growth and development and a nutrient soil eminently adapted to their requirements for existence and reproduction, that from this point occurs the evolution of the disease to neighboring or remote regions.

He combats pathologically the theory of sebaceous gland hypersecretion of pityriasic scales being dried sebum, on the ground that the sebaceous gland ducts are blocked up, instead of being open, and freely discharging their contents, as has been asserted; and that they are in the latter condition only with complete baldness, when the previous destination of the natural oil of the hair had been removed; and that a fat such as sebum does not solidify into scales or crusts but remains fluid. He attributes the fat excess to the coil glands and not to the sebaceous glands. Similar observations upon stenosis of the sebaceous ducts in the affection under consideration have been made by Piffard, Van Harlingen, George T. Elliot and others.

Accepting Unna's postulate that we have to do, not with a stearrhea, a hypersecretion, but with a catarrhal inflammation of the scalp, a parasitic dermatitis, as clinically correct, we have the cue to the direction of appropriate and rational methods of treatment and can thus remove in part the opprobrium of empiricism. We shall see how the disease responds to antiparasitic treatment and how that condition which successfully resisted the best efforts at treatment with the so-called hair tonics and washes disappears under the employment of parasiticides and reducing agents. A brief clinical description of pityriasis capitis may not be inappropriate. The scalp is the seat of a dry, branny desquamation of white scales which are seen among the hairs on the scalp or have fallen upon the coat collar or the clothing. The desquamation is most marked on the vertex and temples. The scales are readily detachable from the scalp and when removed the integument is either normal in appearance or somewhat reddened. There is more or less loss of hair nearly always present. The thinning is most marked at the temples, so that at the forehead there is a jutting peninsula apparent from the lateral recession of the hair and clearing off of the scalp on either side, and also on the vertex or summit of the head where the hair currents produce a sort of whorl or vortex. The amount of alopecia varies within wide limits, and may produce only a trifling thinning, of a greater or smaller tonsure or complete baldness; and may be gradual or rapid. The hair itself is brittle, dry and lustreless and readily falls or is pulled out.

Subjectively, there is usually more or less itching, especially after freely perspiring, and at times there is a soreness and tenderness of the scalp. I shall not go further into the clinical account of the affection, as it is already so well known as to preclude any possibility of a mistake in diagnosis.

I have said nothing of that affection of the scalp characterized by the presence of large, greasy accumulations of scales or adherent crusts, and where the hairs are stuck together and have the look of having been anointed, and where the skin of the forehead, nose and temples are oily and hyperemic, for this is too obviously seborrheic eczema to warrant a separate description. It is plain from the standpoint of the bacterial origin of pityriasis capitis that the ordinary treatment with alkaline soaps, borax, ammonia, etc., is insufficient if not actually harmful. Under their use there is an apparent improvement, as the scales are removed and the supplemental employment of oils and brilliantines gives a certain lustre to the hair, but this improvement is not genuine as the disease manifests itself anew immediately upon the discontinuance of such treatment. The rational indications for treatment lead to the selection of such agents as are anti-parasitic in action and have a reducing effect upon the superficial layers of the epidermis; such are sulphur, resorcin and ichthyol; the main reliance is to be placed on sulphur. The effect of long continued application of sulphur to the unbroken integument is to produce an eruption resembling a typical acute eczema. There is a diffuse hyperemia of the papillary bodies, a dryness and grayness of the horny layer, and the development of isolated, itching papules and vesicles, which subsequently become confluent to produce an eczema rubrum.

It does not cause injury to the deeper layers of the skin nor necrosis of the epidermal cells. It is therefore excellently well adapted for experimental purposes to produce a superficial catarrh upon the skin of lower animals. For the reason that it developed such lesions, Hebra discontinued its use and placed it solely among the remedies for prurigo, scabies and psoriasis.

It is for the reason that a stimulating, reducing and antiparasitic remedy is required that sulphur is used in pityriasis capitis, and it is by far the most efficacious remedy that we possess.

We can choose between three ways of administering it, and may use a lotion, a powder, or a pomade.

The lotion is best adapted to cases of pure seborrheic eczema of the scalp and may be given along with water and alcohol, as in the following formula:

℞ Sulph. precip........................15—30 grammes.
Spts. vini rectif.........................25—30 grammes.
Glycerine pur.......................... 5—10 grammes.
M. Aq. dest.................................. 250

The lotion has the objection that it leaves a heavy yellow deposit upon the scalp and causes some disfigurement. The mode of application is as follows: The head is washed carefully and a small quantity of the lotion is sucked up in a pipette and applied to the middle line of the scalp, so that it may run in all directions. Should the scalp protest against the lotion by becoming irritated, a powder or a pomade had best be substituted. When the patient objects to having the head dampened every night, the following powder may be used with good effect.

℞ Acid salicyl................................ 1 gramme.
Sulph. sublimate...........................12 grammes.
Sod. borat.................................. 5 grammes.
Pulv. talc...................................10 grammes.
M. Pulv. amyli...............................70 grammes.

Or any white and bland powder can be used, such as calcined magnesia, subnitrate of bismuth, oxide of zinc.

The pomades are indicated when the hair is very dry and lustreless and as this is generally the case, they may be more often used than either the lotion or the powder.

The sulphur may be incorporated with vaseline or the following formula of Vidal may be used:

℞ Sulph. precip.............................. 6 grammes.
Ol. theobrom................................10 grammes.
Ol. ricini...................................50 grammes.
M. Bals. peru, vel. tinct benzoin............... 1 gramme.

Bestner and Doyan recommended a pomade consisting of:

℞ Acid salicyl.,
Resorcin, āā................................ 1 gramme.
Bals. peru.................................. 1 gramme.
Sulph...................................... 5—10 grammes.
Vaseline, vel. lanolin.......................50 grammes.
M. A small quantity is rubbed in at night and washed off in the morning.

The simplest pomade is that used by Unna, of Hamburg, and I am able from personal experience, while acting as his assistant, to testify to its value.

It consists of precipitated sulphur ten grammes, pomade ointment (German pharmacopœia) or pomade vaseline, and its mode of employment is the following: In the beginning the application is made every evening. The hair is separated first in the sagittal, then in the coronal direction, the parts being about one centimeter distant from each other. A small amount of the pomade is then taken on the tip of the finger and rubbed into the scalp along these parts, so that the hair may not become stuck together by the ointment. Every three or four evenings the head is washed with Hebra's Spiritus Saponatus Kalinus to clear away the scales and what remains of the ointment. If the desquamation rapidly diminishes, the inunction is done every evening, then every fourth evening, and finally once a week, keeping pace with the improvement in the scalp. The patients are instructed to carefully observe the daily loss of hair, whether there be any increase or diminution. There is apt to be an increase at first, which, however, is temporary, and is occasioned by the loosening of those hairs which were already loosened from the papilla and existed in the follicle only as a foreign body. There is no loss of the papillary hairs. By painstaking and assiduous use of the sulphur preparation the morbid catarrhal process lying at the base of pityriasis capitis will improve in a short time, and an ultimate cure may be confidently expected. If the alopecia has gone on to complete baldness the prognosis is less favorable, yet the brilliant results which occasionally attend treatment serve to render it not absolutely hopeless. In such a condition sulphur alone is inappropriate, but must be used in connection with energetic stimulation by other means.

56½ *Whitheall Street.*

